# Structure and Magnetic Properties of Intermetallic Rare-Earth-Transition-Metal Compounds: A Review

**DOI:** 10.3390/ma15010201

**Published:** 2021-12-28

**Authors:** Lotfi Bessais

**Affiliations:** Department of Physics, University Paris Est Creteil, CNRS, ICMPE, 2 Rue Henri Dunant, F-94320 Thiais, France; bessais@icmpe.cnrs.fr; Tel.: +33-1-4978-1197

**Keywords:** rare-earth-transition-metal intermetallics, mechanical alloying, hard magnetic materials, DFT, Mössbauer spectrometry

## Abstract

This review discusses the properties of candidate compounds for semi-hard and hard magnetic applications. Their general formula is $R_{1-s}T_{5+2s}$ with *R* = rare earth, *T* = transition metal and 0≤s≤0.5 and among them, the focus will be on the ThMn${}_{12}$- and Th${}_{2}$Zn${}_{17}$-type structures. Not only will the influence of the structure on the magnetic properties be shown, but also the influence of various *R* and *T* elements on the intrinsic magnetic properties will be discussed (*R* = Y, Pr, Nd, Sm, Gd, … and *T* = Fe, Co, Si, Al, Ga, Mo, Zr, Cr, Ti, V, …). The influence of the microstructure on the extrinsic magnetic properties of these *R*–*T* based intermetallic nanomaterials, prepared by high energy ball milling followed by short annealing, will be also be shown. In addition, the electronic structure studied by DFT will be presented and compared to the results of experimental magnetic measurements as well as the hyperfine parameter determined by Mössbauer spectrometry.

## 1. Introduction

Rare-earth (*R*) and transition-metal (T,T= Co, Fe, Ni) based magnetic intermetallic compounds (IMC) have several key properties. These properties are divided into two categories: the intrinsic properties and the extrinsic properties. The intrinsic properties are the saturation magnetization ($M_{S}$), the Curie temperature ($T_{C}$), and the magnetocrystalline anisotropy ($K_{u}$). For the herein studied IMCs, these properties are determined by the crystallographic structure and the chemical composition, whereas the extrinsic properties are the coercivity $H_{C}$ and the remanent magnetization $M_{R}$, which are mainly a function of the microstructure.

The IMCs’ magnetic properties determine their potential application. Intermetallic compounds of the *R*–*T* type are formed with all rare-earths (*R*), except lanthanum for $R_{2}$Fe${}_{17}$. These compounds are characterized by a rather low Curie temperature ($T_{C}$), which limits their field of application [1,2]. As generally observed for *T*-rich compounds, the low value of this temperature is attributed to *T*–*T* distances that are too short, particularly for *T* atoms that occupy the positions of the *T*–*T* dumbbells. For short distances between the *T* atoms, antiferromagnetic interactions occur instead of ferromagnetic interactions. As a result, considerable magnetic energy is stored, which leads to the lowering of the $T_{C}$ values.

In addition, the magnetocaloric effect, around the Curie temperature, in some *R*–*T* intermetallic compounds is promising and has been actively studied in recent years [3,4,5].

The magnetocrystalline anisotropy of these compounds is governed by the combined effects of the anisotropy of the rare earth and transition metal atoms. In binary compounds, the rare earth anisotropy is very small at room temperature even though the rare earth sub-lattice has a uniaxial contribution to the anisotropy [6]. It is not sufficient to counteract the anisotropy of the transition metal sub-lattice at room temperature, which favors planar anisotropy.

In order to improve the magnetic properties, partial substitutions of the transition metal must be made and light elements, such as hydrogen, nitrogen or carbon, can be inserted in interstitial positions of the unit cell. The increase in $T_{C}$ and the change in anisotropy from planar to uniaxial opens the way for the use of these compounds in the field of permanent magnets or high-density magnetic recording. However, in addition to high-performance intrinsic properties, it is necessary to optimize the extrinsic properties (coercivity $H_{C}$, remanent magnetization $M_{R}$) by building a suitable microstructure corresponding to the envisaged applications. The microstructure must allow reaching the highest possible coercivity and remanent induction and a magnetization loop close to a rectangular one. The achievement of each of these objectives is facilitated by the existence of a granular microstructure [2,6,7,8,9,10,11,12,13,14,15,16,17,18,19,20,21,22,23,24,25,26,27,28,29,30,31,32,33,34,35,36].

The IMC can be magnetically hard, semi-hard, or soft. Hard intermetallics are characterized by their large hysteresis. They are used as permanent magnets. Semi-hard ones are used for high-density magnetic recording, while soft ones with a very small hysteresis are utilized in electromagnetic machines [37,38].

**Figure 1:**
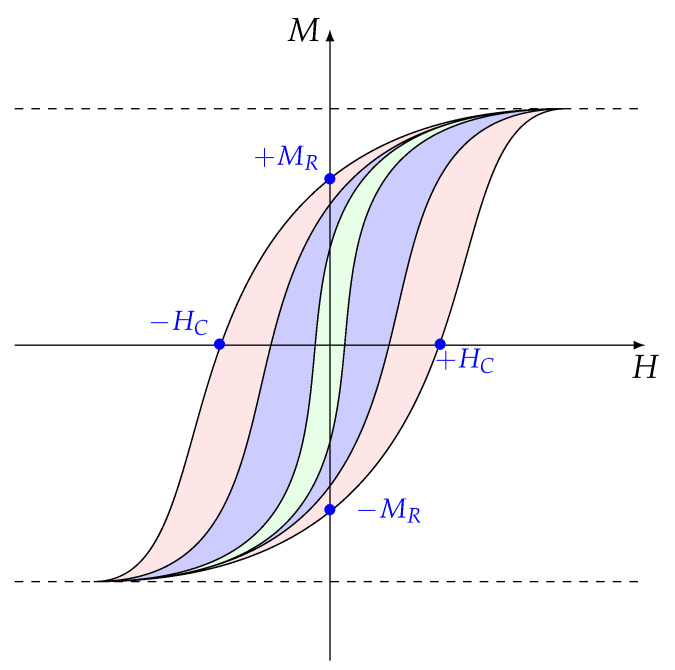
Hysteresis loops for hard (light red), semihard (light blue), and soft (light green) magnets. *M* the magnetization, *H* the applied magnetic field, $M_{R}$ remanent magnetization, and $H_{C}$ the coercivity.

Figure 1 shows a simple approach to distinguish a hysteresis loop from a soft, semihard, or hard magnets, based on the coercivity $H_{C}$ alone. A very wide loop has a high coercivity, while a narrow loop has a very low coercivity, and a semi-hard cycle has an intermediate coercivity. However, Coey has recently shown that we can define a coefficient, called hardness parameter κ, that determines more precisely the difference between these three categories of magnetic materials [38]. $\kappa =\sqrt{K_{u}/mu_{0}M_{S}^{2}}$, where $K_{u}$ is the anisotropy constant, $M_{S}$ is the saturation magnetization, and $\mu_{0}$ the vacuum permeability. For κ<0.1 the magnet is soft, for κ>1 the magnet is hard, and semihard magnets exhibit κ<1.

Recently, Kovacs et al. [39] have clearly shown that one cannot expect a coercive field greater than 1 T in most rare-earth-free magnets. For this reason, the IMCs presented in this review are exclusively based on rare-earths and, more specifically, based on CaCu${}_{5}$-type structure.

To enhance our understanding of *R*-*T* intermetallics for magnetic applications, we gather and discuss, in this review paper, the main achievements made in this field up to now. In particular, the influence of the structure and composition on the intrinsic magnetic properties will be discussed, as well as the effect of the synthesis methods on the extrinsic magnetic properties. A comparison between electronic properties determined by DFT and hyperfine parameters measured by Mössbauer spectrometry will also be shown.

## 2. Overview of Synthesis Methods for Improved Magnetic Properties

### 2.1. Methods for Synthesis of Intermetallic Compounds

Conventional techniques for the synthesis of intermetallic compounds consist of the arc or induction melting of pure elements with subsequent annealing. However, in material science, it is often observed that the synthesis method has a significant influence on the compound’s properties. For the herein discussed compounds, it especially affects the microstructure but also the crystal structure, both of which influence the magnetic properties, as explained in the Introduction. Therefore, the choice of the synthesis method is a non-negligible key parameter.

In the example of *R*–*T* intermetallic compounds, it is often observed that conventional melting methods lead to polycrystalline alloys [40], whereas other techniques such as melt-spinning result in nanocrystalline alloys [41,42]. High-energy ball milling (HEBM) followed by annealing is another possible technique for the production of nanocrystalline alloys and it is well suited to the case of alloys based on rare-earth atoms, extremely volatile elements, because the reaction takes place below their melting temperature [43,44,45,46,47,48]. The annealing times of the powders can be very short, due to the their high reactivity of the nanometric crystallites obtained after HEBM. Moreover, this technique has the advantage of leading to large and homogeneous quantities of materials with reproducible characteristics. For these reasons, our group have chosen to produce *R*–*T*–*M* (*T* = Fe, Co, and *M* = Si, Al, Ga, Mo, Zr, Cr, Ti, V, …) alloys by HEBM followed by annealing.

The powders of *R*, *T*, and *M*, are co-milled in a FRITSCH P7 planetary mill in hermetically sealed jars under a high purity argon atmosphere. The milling is performed in two steps: low energy grinding for half an hour followed by a HEBM for 5 h [44]. The obtained nanocrystalline powders are annealed for 30 min in sealed silica ampoules under a secondary vacuum. The annealing was performed at temperatures between 700 and 1100 ${}^{\circ }$C depending on the desired phase, the out-of-equilibrium phase or the equilibrium one. As an example, for the equilibrium $R\bar{3}m$ Sm${}_{2}$Fe${}_{17}$ phase the chosen temperature is 1050 ${}^{\circ }$C, and to obtain the out-of-equilibrium P6/mmm SmFe${}_{9}$ phase, the annealing temperature is 700 ${}^{\circ }$C.

### 2.2. Methods for Insertion of Light Elements in the Intermetallics

As mentioned in the Introduction, the insertion of light elements (i.e., H, N, C) in the crystal lattice is crucial for the optimization of the intrinsic magnetic properties.

Coey and Sun [8] introduced nitrogen into Sm${}_{2}$Fe${}_{17}$ by heating ground powder in 1 bar of N${}_{2}$ gas. After HEBM, S. V. Veselova et al. performed nitrogenation heat-treating the milled powder with a pressure equal to 40 atm at 450 ${}^{\circ }$C for 24 h in the pure nitrogen atmosphere. Samples were nitrogenated with 2.4 nitrogen atoms per formula unit [49].

Phejar et al. have successfully prepared La(Fe,Si)${}_{13}$H${}_{y}$ hydrides by solid/gas reaction with a Sievert apparatus. The powder was introduced into a container, and then plugged to the hydrogenation apparatus. The sample was then submitted to a hydrogen pressure [44].

Unlike hydrides and nitrides, which were obtained exclusively by solid-gas reaction, carbides were elaborated by different methods:

(i) conventional fusion of appropriate quantities of samarium, iron, and gallium with a Fe-C pre-alloy under argon atmosphere with an excess of samarium [50,51];
(ii) a solid-gas reaction of the Sm${}_{2}$(Fe,*M*)${}_{17}$ alloy (obtained by conventional fusion) with a gaseous hydrocarbon [52,53];
(iii) high-energy ball milling from metallic elements, and reaction with a gaseous hydrocarbon [54].

Compared to nitrides and hydrides, carbides exhibit a greater thermal stability [55,56]. The insertion of the carbon in the elementary unit cell is achieved by a carbonation technique which involves a solid-solid type reaction [57,58,59]. The starting powders, Sm${}_{2}$(Fe,*M*)${}_{17}$, are finely ground, sieved (grain size less than 32 μm) and carefully mixed with the anthracene powder (C${}_{14}$H${}_{10}$) under high purity argon atmosphere. This technique consists in reacting the powder of Sm${}_{2}$Fe${}_{17-x}M_{x}$ compounds with anthracene, also in powder form, in stoichiometric proportion. Each mixture is annealed, in a sealed silica ampoule under a secondary vacuum, for 24 h. The anthracene decomposes releasing hydrogen gas which is fixed by small pieces of magnesium previously placed in the ampoule and separated from the powder by silica wool. At the same time, the temperature was chosen so as not to modify the microstructure of the initial non-carbide alloys [44]. The carbon atoms, thus liberated, then diffuse in the compound according to the reaction:

$$7R-T+\frac{1}{2}C_{14}H_{10}+\frac{7}{2}\mathrm{Mg}⟶7R-T-C+\frac{5}{2}{\mathrm{MgH}}_{2}.$$

## 3. Structure Analysis

**Figure 2:**
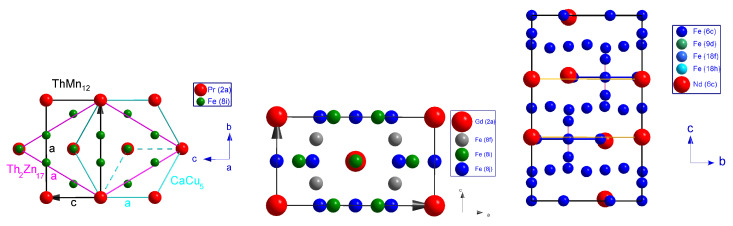
(**Left**) Schematic representation of the relationship between CaCu${}_{5}$ (along [001] axis) Th${}_{2}$Zn${}_{17}$ (along [001] axis) and ThMn${}_{12}$ (along [010] axis) structures, (**Center**) illustration of atomic arrangements in GdFe${}_{10}$Cr${}_{2}$ crystal structure along [010] axis, and (**Right**) the rhombohedral $R\bar{3}m$ crystal structure of Nd${}_{2}$Fe${}_{17}$ along [100] axis.

A large number of rare earth-transition metal-based intermetallic alloys, whether equilibrium or non-equilibrium alloys are derived from the CaCu${}_{5}$-type structure of space group P6/mmm, for example, SmCo${}_{5}$ compound (Table 1). This structure is characterized by a crystallographic site (1a) for the rare earth atom at position (0,0,0) and two distinct sites for the transition metal atoms (2c) at position (1/3,2/3,0) and (3g) at position (1/2,0,1/2). The structure is shown in Figure 2. Over-stoichiometric cobalt equilibrium compounds of the *R*Co${}_{5}$ phase have been studied previously. This results in a slight modification of the lattice due to the presence of cobalt atoms substituted for rare-earth atoms [60]. The general formula of these compounds becomes $R_{1-s}$Co${}_{5+2s}$[29,61,62], where a fraction *s* of the rare earth atoms is randomly replaced by pairs of Co atoms (dumbbell) parallel to the *c* axis. The deviation from the 1/5 stoichiometry strongly influences the magnetic properties of these compounds. For a higher stoichiometry deviation, (s=0.22), the structure has been described as TbCu${}_{7}$ [63,64,65,66].

For specific values of *s*, the substitution becomes ordered and gives rise to derived crystal structures, among which the well-known equilibrium structures ThMn${}_{12}$ (s=0.5), Th${}_{2}$Ni${}_{17}$, and Th${}_{2}$Zn${}_{17}$ (s=0.33). For the latter, one *R* atom out of three is substituted in an ordered manner by an Fe–Fe pair. Figure 2 shows the relationship between CaCu${}_{5}$, ThMn${}_{12}$ and Th${}_{2}$Zn${}_{17}$ structures. In this case, the precursor phase belongs to the P6/mmm space group. For the Sm–Fe binary system, the SmFe${}_{5}$ phase does not exist and the stoichiometry of the Sm${}_{2}$Fe${}_{17}$ phase precursor has been described as SmFe${}_{9}$[23,29] and, very recently, using synchrotron resonant diffraction (SOLEIL), the stoichiometry of this phase has been definitely found as SmFe${}_{8.5}$[62] whereas it was considered in previous publications as TbCu${}_{7}$ [63,64,65,66,67,68,69,70,71,72].

Moreover, D. B. de Mooij and K. H. J. Buschow have demonstrated [73] that the *R*Fe${}_{12}$ binary compound cannot exist. Therefore, a third element *M* (*M* = Cr, Al, Ti, Mo, Si, W, or V) was required to stabilize this phase by forming a ternary *R*Fe${}_{12-x}M_{x}$.

The TbCu${}_{7}$ designation (phase that can exist) attributed to the SmFe${}_{12}$ [65,67,72] (phase that cannot exist [73]) adds an additional difficulty to understand the structure of these non-equilibrium phases.

A recent study [62] had the objective to find the structural relation between 2/17 and the non-equilibrium phase 1/9. On the other hand, this study removes the ambiguity that has often existed for the relationship between the hexagonal TbCu${}_{7}$ phase and the hexagonal P6/mmm phase precursor of the rhombohedral $R\bar{3}m$ phase.

### 3.1. Th${}_{2}$Zn${}_{17}$-Type Structure

**Figure 3:**
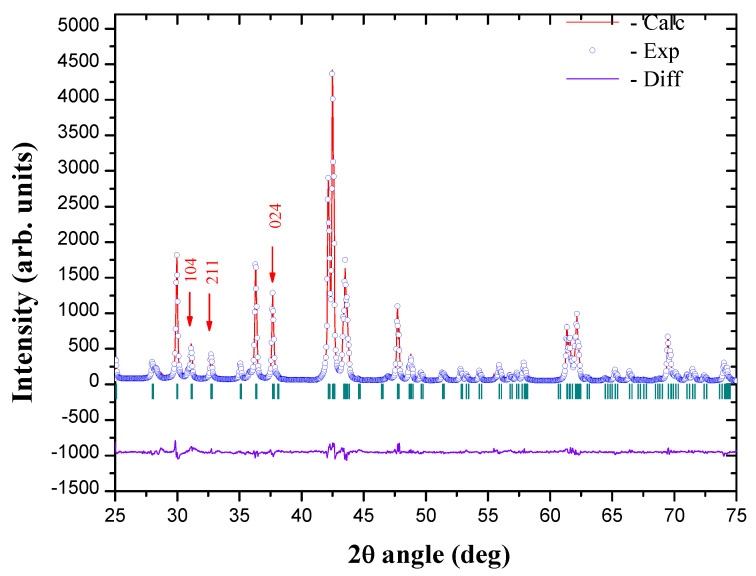
Rietveld refinement of XRD for nanocrystalline Sm${}_{2}$Fe${}_{16}$Ga intermetallic.

The angular positions of the main diffraction peaks, Sm${}_{2}$Fe${}_{17-x}M_{x}$, show a crystallographic structure characteristic of the 2/17 phase of space group $R\bar{3}m$ (Figure 3). The refinement of the X-ray diffraction patterns (XRD) of Sm${}_{2}$Fe${}_{17-x}M_{x}$, is performed by the Rietveld method with the FULLPROF calculation code. This method also allows to determine the size of self-consistent diffraction domains. In the case of Sm${}_{2}$(Fe,*M*)${}_{17}$ compounds, the Rietveld refinement has been performed with the iron distributed on the four sites: 6*c*, 9*d*, 18*f*, 18*h* and the rare earth in site 6*c* (Table 2). These different crystallographic sites of the 2/17 rhombohedral phase are illustrated in Figure 2.

As for the site occupied by *M* atoms, from the Mössbauer spectra explained later, we clearly exclude the occupation of the 6*c* sites (external shoulder on the positive velocity side, characteristic of the total occupation of the *c* site by iron). Site 9d being the smallest among the four sites 6c, 9d, 18f and 18h, was also excluded considering the high radius of *M* = Ga compared to Fe ($r_{\mathrm{Fe}}$ = 1.26 Å, $r_{\mathrm{Ga}}$ = 1.41 Å) [74]. In these conditions there remain two options: occupation of the site 18h or 18f. We clearly see a deterioration of this factor when gallium gradually occupies the 18*f* site. We therefore conclude that gallium is localized at 18*h*, which is in good agreement with the crystallographic results obtained by Teresiak et al. [24]. The structural results of XRD Rietveld refinement are presented for all compositions in Table 3.

The unit cell parameters, *a* and *c*, as well as the volume increase with *x* in a linear way. This increase is due to the substitution of iron atoms by *M*, an element of greater atomic radius than iron. The size of the self-consistent diffraction domains is 60 nm for *x* = 0.5. Moreover, the Rietveld analysis allowed us to quantify the mass percentages of the phases present in the processed samples.

### 3.2. ThMn${}_{12}$-Type Structure

RFe${}_{12}$ binary systems do not exist for any rare earth. Indeed, the atomic radius of iron is smaller than that of manganese in ThMn${}_{12}$, in addition, according to the Pauling classification of elements, iron has eight valence electrons against seven for manganese with a difference of electronegativity higher for iron compared to that with manganese. This induces a high electron density for the 8i site making the alloy unstable.

The reasons why this structure is unstable are therefore: interatomic distances that are too small and a very high electron density provided by iron. However, it is possible to stabilize the 1/12 structure by partially substituting iron with a third element of larger atomic radius; thus a 3d metal element or with a *p*-block element, this leads us to consider the chemical elements that are located to the left of iron in the periodic table.

Some authors report the obtaining of the SmFe${}_{12}$ phase in thin films by non-equilibrium methods such as sputtering [77,78]. However, the obtained XRD pattern shows a simplified line system compared to that corresponding to the 1/12 phase. The explanation given by these authors is the strong texturing of the film along direction (002) obtained along the *c* axis. A refinement by the least square method was carried out, but adopting three different structures which all derive from the structure CaCu${}_{5}$ structure; the ThMn${}_{12}$, the Th${}_{2}$Zn${}_{17}$ (2/17) and also the disordered hexagonal structure TbCu${}_{7}$. The obtained lattice parameters are far from those related to the 2/17 phase. The observation of diffraction patterns does not clarify the nature of the phase, which remains ambiguous, since no indexation is provided but only a suggestion that the phase 1/12 is the closest.

The 1/12 phase could be stabilized by several elements such as Ti [25,79,80,81,82,83,84], Si [80], Mo [79,80,85], W [73,79,80], Cr [79,80,86], V [79,83,87], Al [80]. The solubility and the range of existence of the RFe${}_{12-x}$*M*${}_{x}$ phase vary from one substituent to another. The interest of these phases lies in their Curie temperature which is relatively high compared to that of the 2/17 alloys. It has been shown [88], by ab-initio calculation, that the solubility of Mo is higher than that of Ti, which is in agreement with experiment [89]. When the M (M = Cr, V, Ti, Mo) atoms substitute the Fe atoms in the 8i site, the cohesive energy decreases in a more significant way than when the M atoms go into the Fe 8j or 8f sites. Therefore, the substituents M preferentially occupy the 8i site. The rare earth occupies the 2a (0,0,0) site. As for the elements Fe and M, they are distributed on three crystallographic sites: 8i (0.36,0,0), 8j (0.27,$\frac{1}{2}$,0) and 8f ($\frac{1}{4},\frac{1}{4},\frac{1}{4}$) see Figure 2. The atomic positions and the symmetry of the different sites are presented in the Table 4.

The works relating to 1:12 phases based on praseodymium and 3d transition metals (Fe, Ti, Mo, V) [90,91] report on the difficulties related to their elaboration. Indeed, the nature of the phases obtained and their relative abundances in the sample are closely linked to the annealing temperature. Thus the PrFe${}_{11}$Ti phase (I4/mmm) is obtained mainly in a very small range of annealing temperatures (80 K) between 1303 K and 1383 K. Below 1173 K the majority phase is the 2:17 phase. On the other hand, above 1383 K a peritectic decomposition leads to the precipitation of α−(Fe,Ti) which considerably reduces the abundance of the 1:12 phase.

The ThMn${}_{12}$ structure derives from the CaCu${}_{5}$ structure and can be schematized by the relation:

$$2\;{RT}_{5}\;-\;R\;+2\;T\;=\;{RT}_{12}.$$

Half of the rare earth atoms of the 1/5 structure are replaced by a *T*–*T* pair at position 8i in the 1/12 structure. The unit cell parameters of the two related structures are described by the following expressions (Figure 2):

$$a\left(1/12\right)=b\left(1/12\right)=\frac{2}{3}c\left(1/12\right)\;;\;a\left(1/5\right)=c\left(1/12\right).$$

The refinement of the XRD patterns allowed us to derive the size of the self-consistent diffraction domains and to quantify the mass fractions of the minority phases present. Structural study of the unit cell parameters were systematically measured with an Si standard. The refinement of the diffraction patterns was performed by considering the iron atoms statistically distributed on the different sites 8i, 8j and 8f and considering the substituent M atoms on the site 8i. The unit cell parameters are given in Table 5. Note also that the ratio c/a is not far from the theoretical value $1/\sqrt{3}$ of the tetragonal structure.

E. Tomey et al. [92] found that, for the RFe${}_{10.5}$Mo${}_{1.5}$ series of compounds, where R goes from Pr to Lu, the lattice parameter *a* decreases as a function of R while the *c* parameter remains quasi-constant. The parameter *a* is governed by the atomic radius of the rare-earth.

## 4. Intrinsic Magnetic Properties

In this section, we do not present exclusively the IMCs prepared by HEBM, since the intrinsic properties are independent of the microstructure, and therefore of the synthesis method.

### 4.1. Curie Temperature

For nanocrystalline intermetallics, the Curie temperature, which represents the ferro-paramagnetic transition, must be relatively high for a permanent magnet or for high density magnetic recording applications. Generally, there are two strategies to increase $T_{C}$, either substituting the iron by another metal, or inserting a light element (C, H, N *…*) in the unit cell of the intermetallic compound.

**Figure 4:**
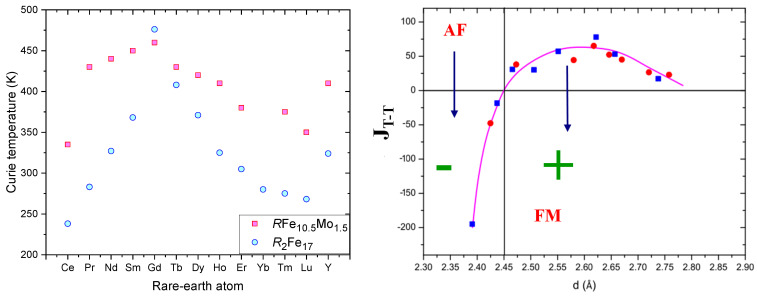
(**Left**) Curie temperatures for different rare-earth atoms, for $R_{2}$Fe${}_{17}$ and *R*Fe${}_{10.5}$Mo${}_{1.5}$[28,30,75,96,97,98]. (**Right**) Exchange interaction $J_{T\text{-}T}$ vs. distances between transition metal atoms (*T*–*T*) for Sm${}_{2}T_{17}$. For interatomic *T*–*T* distances less than 2.45 Å the exchange interaction ($J_{T\text{-}T}$) is negative, while $J_{T\text{-}T}$ is positive in the opposite case [94].

Figure 4 (Left) shows the dependence of Curie temperature on the rare-earth atom for the *R*${}_{2}$Fe${}_{17}$ structure. This behavior is due mainly to the variation of the de Gennes factors of the different rare-earth atoms. The Curie temperature variation for the different rare-earth atom resembles that of the magnetic hyperfine field as we will see later.

The Curie temperatures of Sm${}_{2}$Fe${}_{17-x}M_{x}$ compounds were measured on sealed ampoule samples under the secondary vacuum with an applied field of 1000 Oe. $T_{C}$ was determined by the minima in dM/dT curves, derived from the magnetization measurements.

In *R*${}_{2}$Fe${}_{17}$ intermetallic compounds, the Curie temperature ($T_{C}$) is low, around room temperature (418 K for Sm${}_{2}$Fe${}_{17}$) [23]. This is mainly due to the short Fe–Fe inter-atomic distances of the dumbbells (6c for the $R\bar{3}m$ Sm${}_{2}$Fe${}_{17}$ structure [94] and 2e for P6/mmm SmFe${}_{8.5}$ [62]), where the Fe atoms are anti-ferromagnetically coupled. This distance attached to the dumbbell sites, less than 2.45 Å, leads to negative Fe–Fe interactions [7,95] (Figure 4 (Right)).

**Figure 5:**
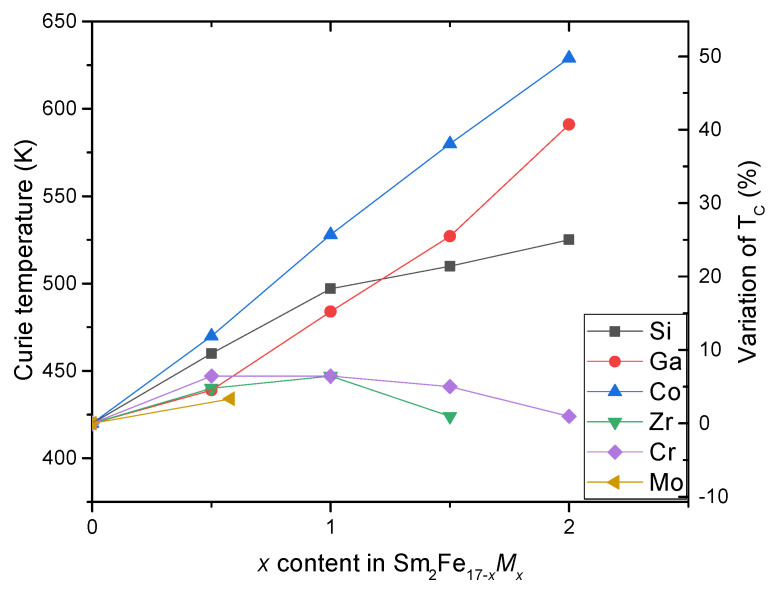
Curie temperature $T_{C}$ and variation $\Delta T_{C}/T_{C}$ of Sm${}_{2}$Fe${}_{17-x}M_{x}$ for *M* = Si, Ga, Co, Zr, Cr, Mo [28,30,75,96,97,98].

With the substitution of *M* atoms in the compounds Sm${}_{2}$Fe${}_{17-x}M_{x}$, the Curie temperature increases monotonically with *x*, then $T_{C}$ decreases (Figure 5, Table 6).

The Curie temperature, for rare-earth-transition-metal compounds, is governed, in general, by three types of interactions:

- 3d–3d ($J_{FeFe}$) exchange interaction between the magnetic moments of the transition-metal atom sublattice;
- 4f–4f($J_{RR}$) exchange between 4f–4f magnetic moments of Sm lattice atoms;
- 3d–4f ($J_{RFe}$) exchange between the two 3d–4f sublattices.

The 4f–4f exchange interaction can be neglected since it is the weakest of the three interactions. We can consider that the only contribution to the Curie temperature is due to the interaction between the magnetic moments of the iron sublattice (3d–3d), if in addition we can also neglect the interactions between the two sublattices (3d–4f) by considering an *R*-Fe system with non-magnetic *R* atom.

In compounds with a rhombohedral structure, the Fe(6c)–Fe(6c) interactions are negative. This is also the case for Fe(9d)–Fe(18f) (weakly negative), while the other Fe–Fe interactions are positive. It was found, for the Sm${}_{2}$Fe${}_{17-x}M_{x}$ compounds, that the $J_{FeFe}$ interaction augments up to *x* = 3. It has also been reported that the coupling constant of the $J_{SmFe}$ sublattices is very small compared to that of $J_{FeFe}$ and it is nearly unrelated to the *M* content. This shows that the Curie temperature is mostly monitored by $J_{FeFe}$. The combination of the two effects, magnetovolume and electronic, can explain the evolution of the Curie temperature. The substitution of non-magnetic elements, such as silicon and aluminum on iron sites in *R*${}_{2}$Fe${}_{17}$ compounds, gives a variation of $T_{C}$ similar to *M* substitution (Figure 5).

### 4.2. Hyperfine Parameters

Mössbauer spectrometry is a particularly recommended technique for samarium-based alloys with an absorption coefficient such that neutron diffraction studies remain very difficult to perform. It allows to confirm the structural results and to determine the hyperfine parameters.

The Mössbauer spectra analysis is based on an adapted and precise simulation method which takes into account the isomer shift (δ). This quantity, which accounts for the density of *s* electrons at the nucleus, is an essential data to understand the effect of the insertion of the light element.

**Figure 6:**
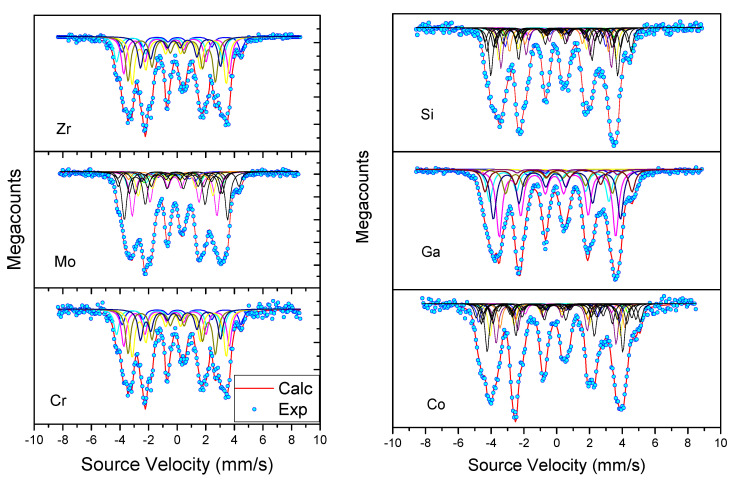
The room temperature Mössbauer spectra of Sm${}_{2}$Fe${}_{16}$*M* (*M* = Zr, Mo, Cr, Si, Ga, Co) [28,30,75,96,97,98].

**Figure 7:**
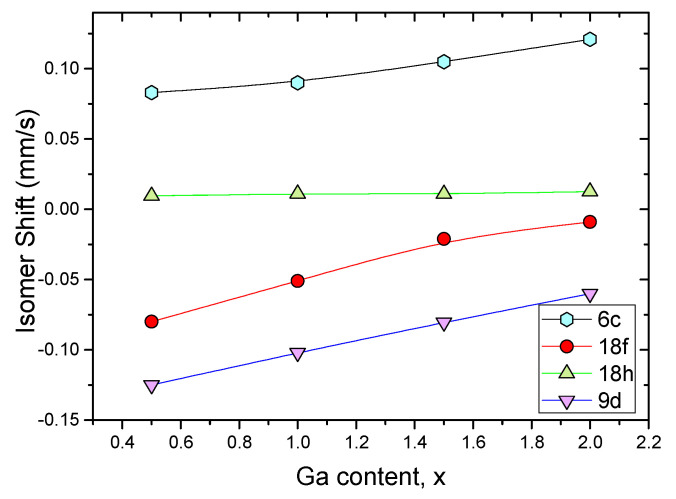
Isomer shift evolution vs substituted Ga atom composition for Sm${}_{2}$Fe${}_{17-x}$Ga${}_{x}$.

**Figure 8:**
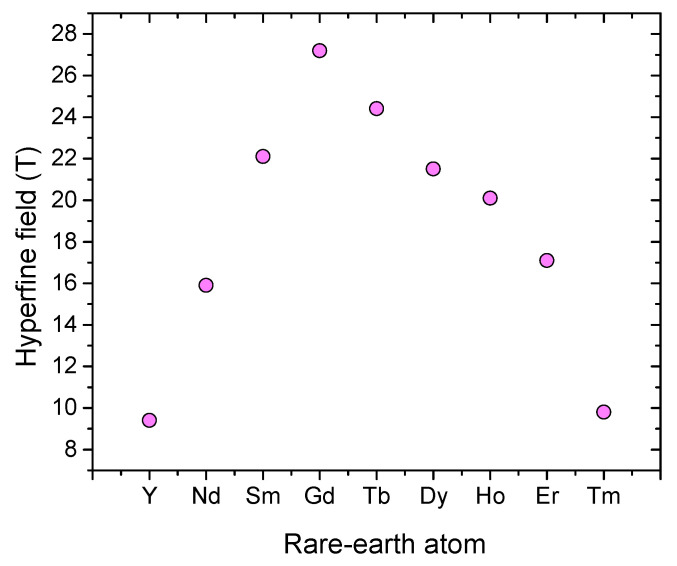
Hyperfine field for different *R*${}_{2}$Fe${}_{17}$ [38,42,99,100,101,102,103,104].

One may be tempted to use a large number of parameters to get a satisfactory simulation. However, the selected solution for the refinement of the Mössbauer spectra must match a physical model supported by several experimental techniques or it could be based on relevant theoretical approaches. The credibility of the suggested model for the fitting of the spectra must be proved by the uniform evolution of the set of hyperfine parameters with a line width slightly higher than the value of 0.25 mm/s, corresponding to the experimental width of the reference α–Fe (Figure 6, Figure 7 and Figure 8).

In addition, the line intensities of the sextuplets were considered in the ratio 3:2:1:1:2:3, assuming a randomly oriented powder, in good agreement with the absence of texture observed on the XRD patterns. A line-width equal to 0.27 mm/s was generally used for each individual sextuplet of the rhombohedral $R\bar{3}m$ Th${}_{2}$Zn${}_{17}$- and the tetragonal I4/mmm ThMn${}_{12}$-type structure. Furthermore, we supposed that the Lamb–Mössbauer absorption factor is the same for all non-equivalent crystallographic sites.

The good resolution of the spectra combined with the crystallographic studies allows a detailed analysis. The refinement of the spectra is then based, on the one hand, on a counting approach of the various magnetic subsites, and on the other hand, on the attribution to these sites of the hyperfine parameters resulting from a good simulation of the spectra for a defined and fixed number of observable sites. Moreover, the evolution of the hyperfine parameters must be coherent and monotonous as a function of the substituted atom rate (Table 7).

The most rigorous method to assign the different sextuplets to the non-equivalent crystallographic sites (6c, 9d, 18f and 18h for Th${}_{2}$Zn${}_{17}$ phase, and 8i, 8j, 18f and 18h for ThMn${}_{12}$ phase) takes into account the hyperfine fields, δ and the Wigner–Seitz cell volumes (WSC). The correlation between the Wigner-Seitz cell volumes and the isomer shift has been established [21,25,82,84,86]. The larger the isomer shift, the larger WSC volume. In addition, the larger the number of iron neighbors of a resonant iron atom, the larger the hyperfine field.

The general approach used for the refinement of the spectra of the different structures will take into account, on the one hand, the correlation between the WSC volumes and δ, and on the other hand, the exact calculation of the abundances of the different sub-sites by means of a binomial distribution law of the substituted atoms.

Mössbauer spectra of the Sm${}_{2}$(Fe,*M*)${}_{17}$ samples (*M* = Ga, Zr, Si, Mo, Cr and Co) recorded at room temperature are shown in Figure 6. These spectra are very complex and magnetically ordered. They result from the convolution of many subspectra; this is due to the existence of the four inequivalent Fe sites, disturbed by the substitution of Fe by the *M* atom at 18h position. The isomer shift of 6c,18f,9d sites slightly increases with the rate of *M* atoms, while that of the site 18h remains constant within the limits of the experimental precision. This particular behavior of the δ{18h} confirms the position of the *M* atom in this specific site.

It should be noted that δ resulting from the sequence of Wigner-Seitz cell calculated volumes: δ{6c}>δ{18h}>δ{18f}>δ{9d} reflect well the perturbations brought by the substitution of iron by the *M* atom, in perfect agreement with the nature of the different environments. Moreover, the evolution of the hyperfine magnetic fields brings an additional argument to the approach we have led.

Figure 8 shows the dependence of hyperfine field ($H_{\mathrm{HF}}$) on the rare-earth atom for *R*${}_{2}$Fe${}_{17}$ structure (Table 7). The change of the $H_{\mathrm{HF}}$ with different rare earth atoms resembles that of the Curie temperature (Figure 4).

**Figure 9:**
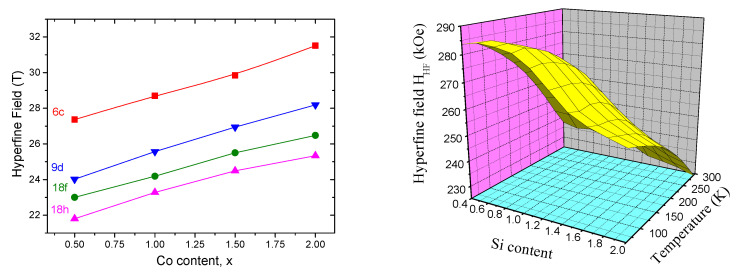
(**Left**) Hyperfine field evolution vs substituted Co atom composition for Sm${}_{2}$Fe${}_{17-x}$Co${}_{x}$. (**Right**) The dependence of hyperfine field on Si content and temperature for Sm${}_{2}$Fe${}_{17-x}$Si${}_{x}$.

The refinement of the hyperfine fields is derived from the analysis of δ since the inequivalent iron site is defined by a set of hyperfine parameters $H_{\mathrm{hf}}$ and δ. It follows that $H_{\mathrm{hf}}\left\{6c\right\}>H_{\mathrm{hf}}\left\{9d\right\}>H_{\mathrm{hf}}\left\{18f\right\}>H_{\mathrm{hf}}\left\{18h\right\}$. Moreover, $H_{\mathrm{hf}}$ strength of a given atom depends on the number of Fe neighbors: the larger the number of Fe neighbors, the higher $H_{\mathrm{hf}}$. The hyperfine field analysis is in perfect agreement with this relationship, site 6c with 13 iron neighbors is by far the largest. Sites 9d and 18f with 10 iron neighbors show average field values, while site 18h with 9 neighbors is the smallest (Figure 9).

Concerning the tetragonal I4/mmm ThMn${}_{12}$-type structure several studies were devoted to *R*Fe${}_{11}$Ti (*R* = Y, Pr, Nd, Sm, Gd) intermetallics [25,84,86,105,106,107]. The refined hyperfine field ($H_{\mathrm{HF}}$), isomer shift (δ), and quadrupole interaction (2ε) for the studied intermetallics are presented in Table 8. For these compounds the following sequence for the hyperfine field was found $H_{\mathrm{HF}}\left\{8i\right\}>H_{\mathrm{HF}}\left\{8j\right\}>H_{\mathrm{HF}}\left\{8f\right\}$, which is coherent with the number of nearest iron neighbors for each inequivalent crystallographic site.

### 4.3. DFT Calculation

The DFT calculation is very useful, not only to calculate the formation energy of intermetallic compounds, but also to determine the theoretical hyperfine field and finally to calculate the total and individual partial magnetic moment per crystallographic site. The objective of the theoretical calculations is to compare the formation energies of the compounds in order to be able to evaluate their stability, and to compare the theoretical and experimental magnetic moments deduced from the Mössbauer spectrometry.

Harashima et al. utilizing first-principal calculations have demonstrated that for any rare-earth *R* atom [108,109] the binary rhombohedral $R_{2}$Fe${}_{17}$ with Th${}_{2}$Zn${}_{17}$-type structure is more stable than the tetragonal *R*Fe${}_{12}$ with ThMn${}_{12}$-type structure. However, if the rare-earth *R* atom (R= Nd or Sm) is partially replaced by Zr, Y or Dy, the difference between the formation energies of 2/17 and 1/12 is lowered.

**Figure 10:**
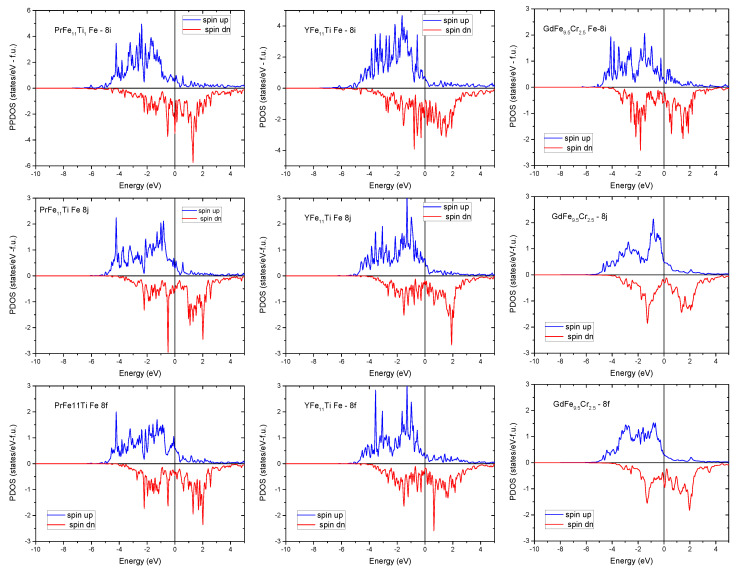
Partial density of states for Fe-8i, Fe 8j and Fe-8f calculated for (**left**) PrFe${}_{11}$Ti, (**middle**) YFe${}_{11}$Ti and (**right**) GdFe${}_{9.5}$Cr${}_{2.5}$. The origin of the energy is located at Fermi energy (black vertical line).

The magnetic moments for the rhombohedral Sm${}_{2}$Fe${}_{17}$ compound was calculated by Yamashita et al. [110] using ab initio approach. The results of these calculations give the following values of magnetic moments for the four inequivalent crystallographic sites: $\mu_{6c}=$ 2.67 $\mu_{B}$, $\mu_{18f}=$ 2.49 $\mu_{B}$, $\mu_{18h}=$ 2.39 $\mu_{B}$, and $\mu_{9d}=$ 2.21 $\mu_{B}$. These values are consistent with those found by Ogura et al. using first-principles electronic structure calculations [111] (Figure 10).

For the tetragonal GdFe${}_{12-x}$Cr${}_{x}$ compound, the DFT calculation allows, among other things, to determine the site of Cr occupation in the I4/mmm space group. The total energy of GdFe${}_{12-x}$Cr${}_{x}$ has been calculated for the three possible substitution Cr sites. This calculation showed that the lowest total energy is consistent with the substitution of iron with chromium in the 8i site (Table 9), indicating that Cr preferentially occupies this site [86]. This result is in agreement with Moze and Buschow’s study of the YFe${}_{12-x}$Cr${}_{x}$ system [112]. It id also in perfect agreement with the Rietveld refinement of the XRD patterns and with the fitting of the Mössbauer spectra of GdFe${}_{12-x}$Cr${}_{x}$ compounds.

Using first-principle calculations, Dirba et al. [113] determined the formation energy and magnetic moments for the three inequivalent sites 8i, 8j and 8f in the SmFe${}_{11}M$, for various *M* elements. The formation energy was calculated as follow:

$$\Delta E=E\left[{\mathrm{SmFe}}_{11}M\right]-\left(E\left[{\mathrm{SmFe}}_{2}\right]+9E\left[\mathrm{Fe}\right]+E\left[M\right]\right).$$

**Figure 11:**
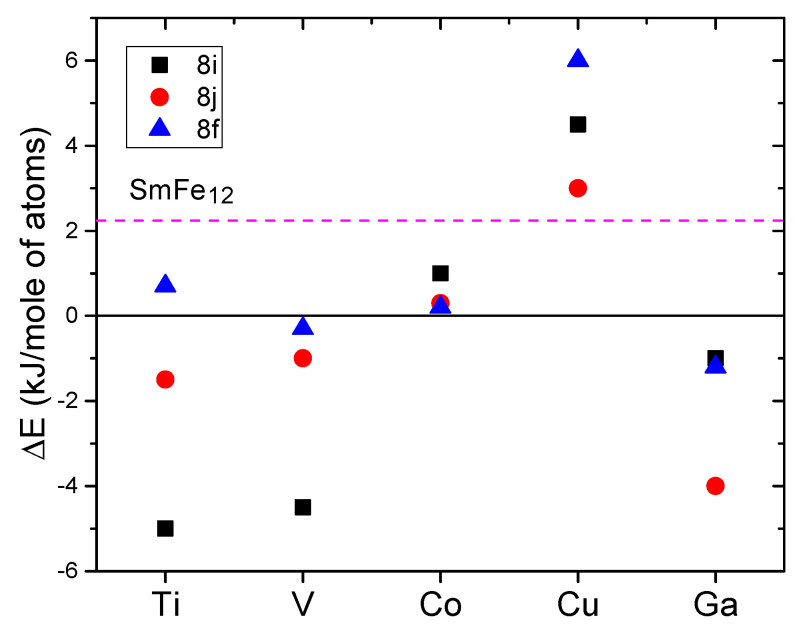
Calculated formation energy for SmFe${}_{11}M$ (*M* = Ti, V, Co, Cu, Ga) [113]. The magenta dashed line corresponds to formation energy of the hypothetical SmFe${}_{12}$ energy.

They found that the substitution of Fe atoms by Ti, V, or Ga enhances the stability of the I4/mmm SmFe${}_{11}$*M* compound (Figure 11), since the calculated formation energies for these elements was found lower than for Co, Cu atoms.

Using ab-initio calculations, Sikora et al. have determined the electronic structure for Gd${}_{0.4}$Tb${}_{0.6}$Co${}_{2}$ intermetallic compound. They have found that cobalt’s magnetic moments, estimated from X-ray photo-electron spectroscopy, are comparable with magnetic moment derived from DFT ab initio calculations [114]. In addition, they showed an anti-parallel alignment between *R* (*R* = Gd, Tb) and Co magnetic moments.

Moreover, we have compared the results of magnetic moments between those obtained by experimental measurements (Mössbauer Spectrometry) and those derived using theoretical ab initio DFT calculations for YFe${}_{11}$Ti, PrFe${}_{11}$Ti, and GdFe${}_{10}$Cr${}_{2}$ compounds. A good agreement have been obtained. These results are summarized in Table 10.

## 5. Extrinsic Magnetic Properties

In this section, we will show the link between microstructure and extrinsic magnetic properties. Before presenting an example of our work on the study of Sm(Fe,Si)${}_{9}$ carbides, we will summarize the most recent works that concern hard magnetic intermetallics prepared by HEBM.

Pal et al. [117] have found that Nd${}_{2}$Fe${}_{14}$B alloy, obtained by HEBM, exhibits high coercivity at room temperature, more than 12 kOe. They concluded that HEBM synthesis method produces effective and less costly as compared to other methods reported earlier. Using HEBM Zhong et al. showed that for the optimized microstructure of Nd${}_{2}$(Fe,Co)${}_{14}$B alloy the coercivity is about 10 kOe with a $M_{S}$ equal to 75 emu/g [118]. Nanoflakes of SmCo${}_{5}$ obtained by HEBM were mixed with Co nanowires in order to obtain SmCo${}_{5}$/Co composites, the highest coercivity obtained is equal to 15 kOe [119]. These 3 examples demonstrate an important aspect: without modifying the structure and/or composition, the values for coercivity can be changed and optimized by adapting the synthesis method.

**Figure 12:**
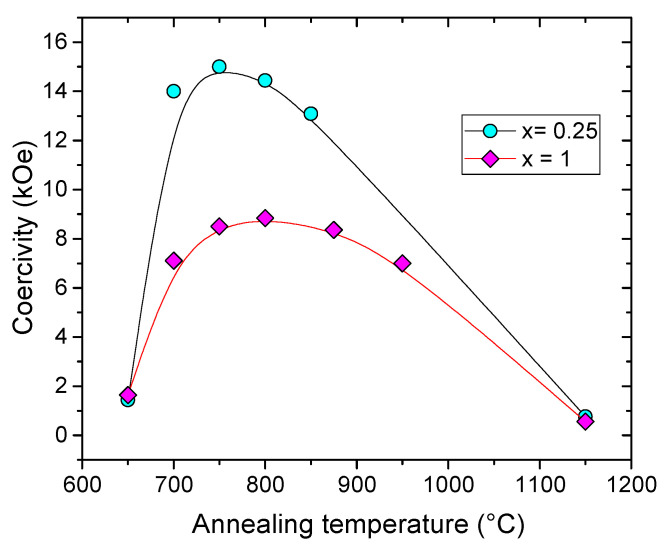
Room temperature coercivity vs annealing temperature for SmFe${}_{9-x}$Si${}_{x}$C.

Through the example of SmFe${}_{9-x}$Si${}_{x}$C carbides, we will see some synthesis parameters that influence the coercivity and explain the reason. The coercive fields measured at room temperature for the nanocrystalline carbide SmFe${}_{9-x}$Si${}_{x}$C compounds (x=0.25,1) as a function of the annealing temperature $T_{A}$ of the HEBM samples before carbonation, shown in Figure 12. For $T_{A}>1000{\;}^{\circ }$C the hexagonal P6/mmm structure turns into rhombohedral $R\bar{3}m$ one. At $T_{A}=750{\;}^{\circ }$C, a maximum of coercive force of 15 kOe was obtained for SmFe${}_{8.75}$Si${}_{0.25}$C compound, while for SmFe${}_{8}$Si${}_{1}$C, which is described by the space group P6/mmm, the maximum of $H_{C}$ seems to move towards 800 ${}^{\circ }$C. The value of $H_{C}$ remains high, close to 13 kOe, for x=0.5 but decreases towards 8.5 kOe for x=1. This evolution can be linked to the variation of the crystallographic unit cell volume. Indeed the volume augmentation under the effect of carbonation decreases with the silicon content. The relative increase in volume ΔV/V varies from 3.70% for x=1 to 5.05% for x=0.25. This effect is, on the one hand, in agreement with the increase of the magnetic moment per iron atom, and on the other hand, it might correspond to a reduction of the magnetic anisotropy field with the increase of the silicon content.

The analysis of the coercivity suggests two different regimes depending on $T_{A}$. A too low annealing temperature slows down the reaction in the solid state leading to the metastable Sm(Fe,Si)${}_{9}$C phase responsible for the magnetic hardening. A higher annealing temperature reduces the number of defects in the P6/mmm phase. This results in an increase of $H_{C}$, but on the other hand, the size of the diffraction domains increases which decreases $H_{C}$.

**Figure 13:**
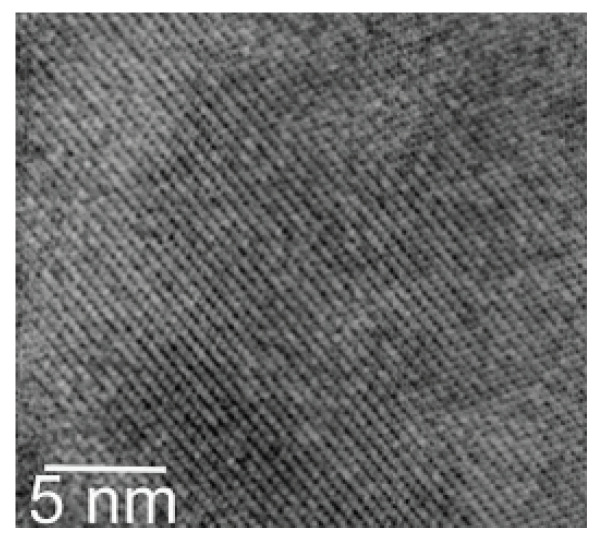
High-resolution Transmission Electron Microscopy (HRTEM) image for the best microstructure corresponding to domain size of 22 nm.

High-resolution transmission electron microscopy (HRTEM) was used to study the morphology of these specific samples (Figure 13). Measurements of inter-reticular distances from fringe system processing offer the possibility to identify grains from their crystallographic parameters. For the SmFe${}_{8.75}$Si${}_{0.25}$C compound, annealed at $T_{A}=650{\;}^{\circ }$C, the grain size is small around 10 nm and $H_{C}$ is equal to 1.5 kOe. For the same sample annealed at a higher temperature (1150 ${}^{\circ }$C), the grain size is around 40 nm and the corresponding coercive field is equal to 0.8 kOe.

For the sample with the best coercive field, 15 kOe, the grain size is on the order of 22 nm in agreement with the Rietveld analysis. It appears that the grain boundaries connect neighboring grains without an inter-granular layer of measurable size, which favors the magnetic exchange coupling between grains. Moreover, a slight increase of the $M_{R}/M_{S}$ ratio equal to 0.6 is observed ($M_{R}$ represents the remanent magnetization and $M_{S}$ the saturation magnetization). This could be explained by the exchange coupling between the grains of the main phase, rather than by the intermediary of the iron grains found in small quantities by crystallographic analysis and Mössbauer spectroscopy. Indeed the same tendency is observed for the $M_{R}/M_{S}$ ratio, for the other compositions which do not contain free iron. The high coercivity of these compounds obtained by HEBM followed by annealing results from the non-equilibrium precursor phase Sm(Fe,Si)${}_{9}$C rather than from the equilibrium compounds Sm${}_{2}$(Fe,Si)${}_{17}$C${}_{2}$.

In addition, nanocrystalline compounds PrCo${}_{3}$, Pr${}_{2}$Co${}_{7}$ and Pr${}_{5}$Co${}_{19}$, constituted of intergrowths of AB${}_{5}$-AB${}_{2}$ stacking blocks, were studied [120,121,122]. Thanks to the optimization of their microstructures we obtained the following coercivities 12 kOe, 18 kOe, 15 kOe for PrCo${}_{3}$, Pr${}_{2}$Co${}_{7}$, Pr${}_{5}$Co${}_{19}$, respectively. More recently, Bajorek et al. have shown a very significant potential for low-cost synthesis method of nanocrystalline hard magnetic SmCo${}_{5}$ compound [48]. They used an innovative wet milling method that allowed them to optimize the microstructure for better extrinsic magnetic properties [43,45,46,47,48].

## 6. Conclusions

In this review, we have presented the structural and magnetic properties of some R-Fe-M-X intermetallics obtained by high energy milling. The combined effect of chemical substitution and light element insertion has been highlighted to optimize the intrinsic magnetic properties. The evolution of the Curie temperature is interpreted in terms of electronic effect and/or magnetovolume effect. The results of the magnetic measurements have been compared to the theoretical ab initio calculations and to the magnetic moments deduced from the hyperfine field measured by Mössbauer spectrometry. Finally, for the nanocrystalline hard magnetic materials, we showed the relationship between the extrinsic magnetic properties and the optimized microstructure of the nanocrystalline intermetallics.

## Figures and Tables

**Table 1:** Atom Wyckoff positions, number of atoms in hexagonal P6/mmm$R_{1-s}T_{5+2s}$ structure. s=0, s=0.33, and s=0.5 correspond to CaCu${}_{5}$, Th${}_{2}$Zn${}_{17}$, and ThMn${}_{12}$ structure, respectively.

Atomic	Occup.	s=0	s=0.03	s=0.33	s>0.33	s=0.5
Position		CaCu${}_{5}$		2/17		1/12
R(1a) 0,0,0	1−s	1	0.97	0.66	1−s	0.5
T(2c) $\frac{1}{3},\frac{1}{2},0$	2(1−3s)	2	1.92	0	0	0
T(6l) x,2x,0	6*s*	0	0.18	2	2	2
T(3g) $\frac{1}{2},0,\frac{1}{2}$	3	3	3	3	3	3
T(2e) 0,0,z	2*s*	0	0.06	0.66	2*s*	1

**Table 2:** Atom and Wyckoff positions for the $R_{2}$Fe${}_{17}$$R\bar{3}m$ rhombohedral structure.

Atom	Wyckoff Position
Sm 6c	(0,0,*z*)
Fe1 6c	(0,0,*z*)
Fe2 9d	($\frac{1}{2}$,0,$\frac{1}{2}$)
Fe3 18f	(*x*,0,0)
Fe4 18h	(*x*,*x*,*z*)

**Table 3:** XRD analysis results obtained by Rietveld method on the Sm${}_{2}$Fe${}_{17-x}$*M*${}_{x}$ (*M* = Si, Ga, Co, Zr, Cr, Mo) and (*x* = 0, 0.5, 1, 1.5, 2) alloys [25,26,27,28,29,30,62,75,76].

Alloy	a(Å)	c(Å)	x(18f)	x(18h)	z(6c)Sm	z(6c)Fe	z1(8h)	V(Å)${}^{3}$	R${}_{B}$	$\chi^{2}$
Sm${}_{2}$Fe${}_{17}$	8.558	12.441	0.291	0.501	0.342	0.094	0.156	789	5.05	1.30
Sm${}_{2}$Fe${}_{16.5}$Si${}_{0.5}$	8.547	12.437	0.291	0.501	0.341	0.095	0.156	787	4.62	2.10
Sm${}_{2}$Fe${}_{16}$Si	8.542	12.436	0.291	0.501	0.342	0.096	0.157	786	6.01	1.80
Sm${}_{2}$Fe${}_{15.5}$Si${}_{1.5}$	8.527	12.428	0.291	0.501	0.342	0.095	0.157	782	4.40	2.40
Sm${}_{2}$Fe${}_{15}$Si${}_{2}$	8.520	12.428	0.292	0.501	0.342	0.096	0.157	781	5.69	2.20
Sm${}_{2}$Fe${}_{16.5}$Ga${}_{0.5}$	8.567	12.457	0.289	0.502	0.343	0.096	0.156	791	4.25	1.40
Sm${}_{2}$Fe${}_{16}$Ga	8.582	12.483	0.290	0.502	0.342	0.094	0.156	796	6.11	2.50
Sm${}_{2}$Fe${}_{15.5}$Ga${}_{1.5}$	8.598	12.510	0.290	0.502	0.342	0.097	0.156	801	5.63	2.70
Sm${}_{2}$Fe${}_{15}$Ga${}_{2}$	8.619	12.540	0.291	0.502	0.342	0.097	0.156	807	5.55	2.50
Sm${}_{2}$Fe${}_{16.5}$Co${}_{0.5}$	8.550	12.450	0.289	0.501	0.343	0.096	0.156	788	4.56	1.61
Sm${}_{2}$Fe${}_{16}$Co	8.548	12.456	0.290	0.502	0.343	0.096	0.157	788	4.73	1.58
Sm${}_{2}$Fe${}_{15.5}$Co${}_{1.5}$	8.545	12.463	0.291	0.501	0.343	0.096	0.156	788	4.05	1.40
Sm${}_{2}$Fe${}_{15}$Co${}_{2}$	8.544	12.467	0.291	0.502	0.343	0.095	0.156	788	3.58	1.76
Sm${}_{2}$Fe${}_{16.5}$Zr${}_{0.5}$	8.541	12.449	0.289	0.502	0.343	0.096	0.157	786	4.53	1.34
Sm${}_{2}$Fe${}_{16}$Zr	8.532	12.465	0.290	0.502	0.343	0.096	0.157	786	5.10	1.42
Sm${}_{2}$Fe${}_{15.5}$Zr${}_{1.5}$	8.527	12.457	0.289	0.501	0.344	0.096	0.157	784	4.33	1.52
Sm${}_{2}$Fe${}_{16.5}$Cr${}_{0.5}$	8.549	12.445	0.289	0.502	0.343	0.096	0.157	787	6.91	1.26
Sm${}_{2}$Fe${}_{16}$Cr	8.542	12.450	0.290	0.502	0.343	0.096	0.157	787	5.75	1.26
Sm${}_{2}$Fe${}_{15.5}$Cr${}_{1.5}$	8.535	12.442	0.289	0.501	0.344	0.096	0.157	785	4.06	1.57
Sm${}_{2}$Fe${}_{15}$Cr${}_{2}$	8.533	12.442	0.291	0.501	0.343	0.092	0.156	784	4.49	1.16
Sm${}_{2}$Fe${}_{16.42}$Mo${}_{0.58}$	8.558	12.472	0.289	0.502	0.342	0.096	0.157	791	4.61	1.10

**Table 4:** Atom and Wyckoff positions for the *R*Fe${}_{11}$Ti I4/mmm tetragonal structure.

Atom	Symmetry	Wyckoff Position
R 2a	4/mmm	(0,0,0)
Fe1 8f	2/m	($\frac{1}{4}$,$\frac{1}{4}$,$\frac{1}{4}$)
Fe2 8i	m2m	(*x*,0,0)
Fe3 8j	m2m	(*x*,$\frac{1}{2}$,0)

**Table 5:** XRD analysis results obtained by Rietveld method on the *R*Fe${}_{12-x}$*M*${}_{x}$ (*M* = Si, Ti, Cr, V, Cr, Mo) and (*x* = 0, 0.5, 1, 1.5, 2) alloys [25,26,27,28,29,30,62,75,76,82,84].

Alloy	a(Å)	c(Å)	Ref.
YFe${}_{11}$Ti	8.503(3)	4.789(4)	[84]
SmFe${}_{11}$Ti	8.555(3)	4.794(3)	[25]
YFe${}_{10.5}$Mo${}_{1.5}$	8.527(2)	4.784(2)	[92]
CeFe${}_{10.5}$Mo${}_{1.5}$	8.535(1)	4.769(2)	[92]
PrFe${}_{10.5}$Mo${}_{1.5}$	8.600(2)	4.785(3)	[92]
NdFe${}_{10.5}$Mo${}_{1.5}$	8.584(1)	4.780(2)	[92]
SmFe${}_{11}$Mo	8.565(2)	4.786(2)	[29]
GdFe${}_{10.5}$Mo${}_{1.5}$	8.553(2)	4.795(1)	[92]
TbFe${}_{10.5}$Mo${}_{1.5}$	8.562(2)	4.782(1)	[92]
DyFe${}_{10.5}$Mo${}_{1.5}$	8.523(1)	4.782(1)	[92]
HoFe${}_{10.5}$Mo${}_{1.5}$	8.518(3)	4.786(3)	[92]
ErFe${}_{10.5}$Mo${}_{1.5}$	8.515(2)	4.783(1)	[92]
LuFe${}_{10.5}$Mo${}_{1.5}$	8.483(3)	4.770(2)	[92]
SmFe${}_{10}$V${}_{2}$	8.528(6)	4.770(3)	[83]
PrFe${}_{11}$Ti	8.594(2)	4.789(2)	[84]
NdFe${}_{11}$Ti	8.579(3)	4.795(2)	[83]
GdFe${}_{11}$Ti	8.533(3)	4.789(3)	[93]
GdFe${}_{10}$Cr${}_{2}$	8.480(6)	4.755(3)	[86]

**Table 6:** Curie temperature of Sm${}_{2}$Fe${}_{17-x}$*M*${}_{x}$ (*M* = Si, Ga, Co, Zr, Cr, Mo) and (*x* = 0, 0.5, 1, 1.5, 2) alloys.

Alloy	$T_{C}$ (K)	References
Sm${}_{2}$Fe${}_{17}$	420	[97]
Sm${}_{2}$Fe${}_{16.5}$Si${}_{0.5}$	460	[96]
Sm${}_{2}$Fe${}_{16}$Si	497	
Sm${}_{2}$Fe${}_{15.5}$Si${}_{1.5}$	510	
Sm${}_{2}$Fe${}_{15}$Si${}_{2}$	525	
Sm${}_{2}$Fe${}_{16.5}$Ga${}_{0.5}$	439	[97]
Sm${}_{2}$Fe${}_{16}$Ga	484	
Sm${}_{2}$Fe${}_{15.5}$Ga${}_{1.5}$	527	
Sm${}_{2}$Fe${}_{15}$Ga${}_{2}$	591	
Sm${}_{2}$Fe${}_{15}$Ga${}_{3}$	596	
Sm${}_{2}$Fe${}_{16.5}$Co${}_{0.5}$	696	[28]
Sm${}_{2}$Fe${}_{16}$Co	707	
Sm${}_{2}$Fe${}_{15.5}$Co${}_{1.5}$	717	
Sm${}_{2}$Fe${}_{15}$Co${}_{2}$	732	
Sm${}_{2}$Fe${}_{16.5}$Zr${}_{0.5}$	440	[76]
Sm${}_{2}$Fe${}_{16}$Zr	447	
Sm${}_{2}$Fe${}_{15.5}$Zr${}_{1.5}$	424	
Sm${}_{2}$Fe${}_{16.5}$Cr${}_{0.5}$	447	[75]
Sm${}_{2}$Fe${}_{16}$Cr	447	
Sm${}_{2}$Fe${}_{15.5}$Cr${}_{1.5}$	441	
Sm${}_{2}$Fe${}_{15}$Cr${}_{2}$	424	
Sm${}_{2}$Fe${}_{16.42}$Mo${}_{0.58}$	434	[29]

**Table 7:** Hyperfine parameters of $R_{2}$Fe${}_{17-x}$.

	$\mu_{0}H_{\mathrm{HF}}\left(T\right)$	Ref
Y${}_{2}$Fe${}_{17}$	9.4(2)	[99]
Nd${}_{2}$Fe${}_{17}$	15.9(1)	[100]
Sm${}_{2}$Fe${}_{17}$	22.1(2)	[38]
Gd${}_{2}$Fe${}_{17}$	27.2(2)	[101]
Tb${}_{2}$Fe${}_{17}$	24.4(1)	[102]
Dy${}_{2}$Fe${}_{17}$	21.5(4)	[42]
Ho${}_{2}$Fe${}_{17}$	20.1(2)	[99]
Tm${}_{2}$Fe${}_{17}$	17.1(1)	[103]
Er${}_{2}$Fe${}_{17}$	9.8(1)	[104]

**Table 8:** Room temperature Mössbauer hyperfine parameters for *R*Fe${}_{12-x}$*M*${}_{x}$: Hyperfine field ($H_{\mathrm{HF}}$), isomer shift (δ) and quadrupole interaction (2ε). 〈HF〉 denotes the average of the hyperfine parameters.

	Fe{8i}	Fe{8j}	Fe{8f}	〈HF〉	Ref.
YFe${}_{11}$Ti					[84]
$\mu_{0}H_{\mathrm{HF}}$(T)	26.8	22.7	20.1	23.2	
δ (mm/s)	−0.09	−0.12	−0.17	−0.13	
2ε (mm/s)	0.08	0.07	0.04	0.06	
PrFe${}_{11}$Ti					[84]
$\mu_{0}H_{\mathrm{HF}}$(T)	27.1	23.8	21.8	24.2	
δ (mm/s)	−0.08	−0.10	−0.12	−0.09	
2ε (mm/s)	0.09	0.09	0.04	0.08	
NdFe${}_{11}$Ti					[105]
$\mu_{0}H_{\mathrm{HF}}$(T)	32.6	29.6	25.2	28.1	
δ (mm/s)	0.01	−0.02	−0.15	−0.09	
2ε (mm/s)	0.03	0.10	0.02	0.08	
SmFe${}_{11}$Ti					[25]
$\mu_{0}H_{\mathrm{HF}}$(T)	27.2	23.5	25.8	28.1	
δ (mm/s)	0.01	−0.02	−0.15	−0.09	
2ε (mm/s)	0.03	0.10	0.02	0.08	
GdFe${}_{11}$Ti					[86]
$\mu_{0}H_{\mathrm{HF}}$(T)	26.9	25.1	23.9	25.1	
δ (mm/s)	−0.06	−0.11	−0.13	0.14	
2ε (mm/s)	0.05	0.04	0.04	0.04	

**Table 9:** Calculated energy *E* (Ry) of GdFe${}_{10}$Cr${}_{2}$, YFe${}_{11}$Cr, YFe${}_{11}$Ti, and YFe${}_{11}$V with Cr, Ti, and V atoms occupying different sites.

Compound	8i	8j	8f	Ref.
GdFe${}_{10}$Cr${}_{2}$	−104,440.2439	−104,440.2112	−104,440.2026	[86]
YFe${}_{11}$Cr	−36,874.7094	−36,874.6956	−36,874.6966	[115]
YFe${}_{11}$Ti	−36,480.6233	−36,480.6114	−36,480.6025	[84]
YFe${}_{11}$V	−36,671.7028	−36,671.6735	−36,671.6745	[116]

**Table 10:** Comparison between magnetic moments, in $\mu_{B}$/at., obtained by experimental measurements (Mössbauer Spectrometry) and by DFT calculations for YFe${}_{11}$Ti, PrFe${}_{11}$Ti, and GdFe${}_{10}$Cr${}_{2}$ [84,86].

	$\mu_{\mathrm{Fe}}\left\{8i\right\}$	$\mu_{\mathrm{Fe}}\left\{8i\right\}$	$\mu_{\mathrm{Fe}}\left\{8i\right\}$	$\langle \mu_{\mathrm{Fe}}\rangle$
YFe${}_{11}$Ti				
Mössbauer	1.98	1.77	1.56	1.77
Calculated	2.49	2.07	1.88	1.90
PrFe${}_{11}$Ti				
Mössbauer	2.00	1.84	1.69	1.82
Calculated	2.53	2.16	1.91	2.12
GdFe${}_{10}$Cr${}_{2}$				
Mössbauer	2.10	2.04	1.98	2.03
Calculated	2.46	2.36	2.12	2.28

## Data Availability

Data available on request due to restrictions e.g., privacy or ethical.

## Abbreviations

The following abbreviations are used in this manuscript:

DFT	Density Functional Theory
δ	Isomer shift
HEBM	High-energy ball milling
HRTEM	High-resolution transmission electron microscopy
$H_{\mathrm{HF}}$	Hyperfine field
$H_{C}$	Coercivity
$M_{R}$	Remanent magnetization
$M_{S}$	Saturation magnetization
R	Rare-earth
$T_{A}$	Annealing temperature
$T_{C}$	Curie temperature
WSC	Wigner-Seitz cell
XRD	X-ray Diffraction
